# Tenofovir Nephrotoxicity: 2011 Update

**DOI:** 10.1155/2011/354908

**Published:** 2011-06-07

**Authors:** Beatriz Fernandez-Fernandez, Ana Montoya-Ferrer, Ana B. Sanz, Maria D. Sanchez-Niño, Maria C. Izquierdo, Jonay Poveda, Valeria Sainz-Prestel, Natalia Ortiz-Martin, Alejandro Parra-Rodriguez, Rafael Selgas, Marta Ruiz-Ortega, Jesus Egido, Alberto Ortiz

**Affiliations:** ^1^Nefrología, IIS-Fundacion Jimenez Diaz, Fundacion Renal Iñigo Alvarez de Toledo/Instituto Reina Sofia de Investigacion Nefrologica (FRIAT/IRSIN), Universidad Autonoma de Madrid, Madrid, Spain; ^2^Medicina Interna, IIS-Fundacion Jimenez Diaz, Madrid, Spain; ^3^Nefrología, IDiPaz, Universidad Autonoma de Madrid, Fundacion Renal Iñigo Alvarez de Toledo/Instituto Reina Sofia de Investigacion Nefrologica (FRIAT/IRSIN), Madrid, Spain; ^4^Unidad de Diálisis, Fundación Jiménez Díaz, Avenida Reyes Católicos 2, 28040 Madrid, Spain

## Abstract

Tenofovir is an acyclic nucleotide analogue reverse-transcriptase inhibitor structurally similar to the nephrotoxic drugs adefovir and cidofovir. Tenofovir is widely used to treat HIV infection and approved for treatment of hepatitis B virus. Despite initial cell culture and clinical trials results supporting the renal safety of tenofovir, its clinical use is associated with a low, albeit significant, risk of kidney injury. Proximal tubular cell secretion of tenofovir explains the accumulation of the drug in these mitochondria-rich cells. Tenofovir nephrotoxicity is characterized by proximal tubular cell dysfunction that may be associated with acute kidney injury or chronic kidney disease. Withdrawal of the drug leads to improvement of analytical parameters that may be partial. Understanding the risk factors for nephrotoxicity and regular monitoring of proximal tubular dysfunction and serum creatinine in high-risk patients is required to minimize nephrotoxicity. Newer, structurally similar molecular derivatives that do not accumulate in proximal tubules are under study.

## 1. Tenofovir

**Figure 1:**
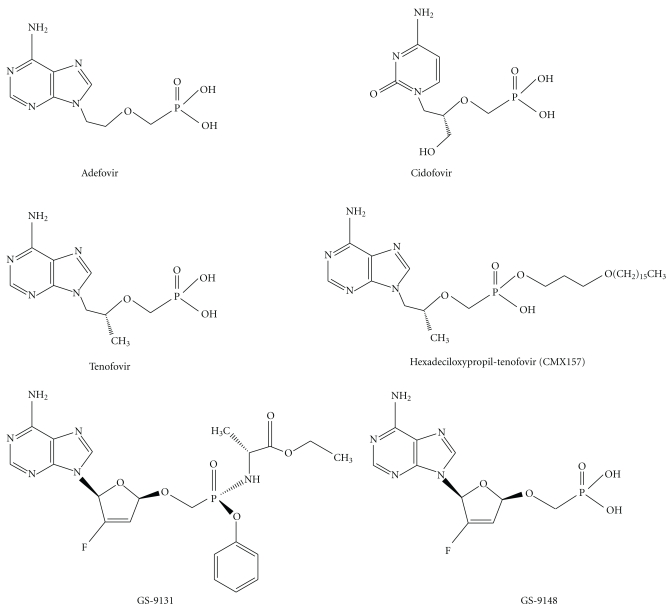
Chemical structure of the three main nephrotoxic acyclic nucleotide analogs, adefovir, cidofovir and tenofovir, as well as less nephrotoxic tenofovir derivatives under development. A lesser uptake by proximal tubular cells can be achieved by either esterifying the compounds with an alkoxyalkyl group, in effect disguising them as lysophospholipids (hexadeciloxypropyl-tenofovir, CMX157) or by ribose-modification (GS-9148 and its oral prodrug GS-9131).

Tenofovir disoproxil fumarate is an orally bioavailable prodrug of tenofovir, an acyclic nucleotide analogue reverse-transcriptase inhibitor (NtRTI) structurally similar to adefovir and cidofovir [1] (Figure 1). Acyclic nucleotides differ in their side chains: hydroxy phosphonomethoxypropyl (HPMP) for cidofovir, phosphonomethoxyethyl (PME) for adefovir and phosphonomethoxypropyl (PMP) for tenofovir [2]. Tenofovir diphosphate is a structural analog of deoxyadenosine-5′-triphosphate, the usual substrate for viral RNA-directed DNA polymerase, and is a weak inhibitor of mammalian DNA *α*- and *β*-polymerases and mitochondrial DNA *γ*-polymerase [3].

Tenofovir was the first (2001), and remains the only, NtRTI approved by the US Food and Drug Administration (FDA) for the treatment of HIV infection [1]. Tenofovir was also approved for treatment of chronic hepatitis B in adults in 2008 [4]. Tenofovir is now a widely used component of antiretroviral regimens for both treatment-naive and experienced patients on the basis of its efficacy and tolerability in clinical trials. US HIV treatment guidelines considered tenofovir as part of all preferred regimens for antiretroviral-naive adults and adolescents [5]. Tenofovir is available in fixed-dose combination with emtricitabine and efavirenz [6]. Up to the end of 2007, the cumulative experience with tenofovir in Europe and North America was around 455 392 person-years [7].

**Figure 2:**
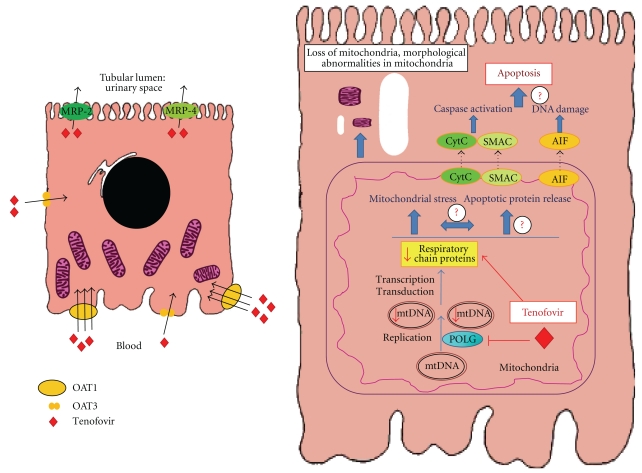
Tenofovir handling by proximal tubular cells and potential molecular mechanisms and clinical consequences of tenofovir nephrotoxicity. (a) Tenofovir secretion by proximal tubular cells: 20 to 30% of tenofovir is excreted unchanged in the urine through active secretion by proximal tubular cells. OAT1 is the main transporter taking tenofovir into the proximal tubular cell, although OAT3 also collaborates. Once inside this mitochondria-rich cell type, tenofovir must be extruded into the tubular lumen by MRP-2 and MRP-4. Blocking tenofovir uptake by OAT1 may protect tubular cells by keeping intracellular tenofovir level low. Thus, probenecid is routinely used to prevent cidofovir nephrotoxicity since cidofovir is also transported into tubular cells by OAT1. There is less experience preventing tenofovir nephrotoxicity. A decreased GFR will increase plasma tenofovir levels and proximal tubular cell uptake through OAT1. Blocking tenofovir extrusion by MRP-2 and MRP-4 by pharmacological interference may also boost tenofovir nephrotoxicity. OAT: organic acid transporter; MRP: multidrug resistance protein. (b) Potential molecular mechanisms of tenofovir toxicity towards proximal tubular cells. Proximal tubular cells are uniquely susceptible to tenofovir toxicity because they gave a complement of transporters that increase intracellular concentrations of the drug, and they are rich in mitochondria. Tenofovir and other acyclic nucleotides decrease mtDNA content by inhibiting mitochondrial DNA polymerase *γ* (POLG). This has been related to structural mitochondrial abnormalities, some of them visible even by optical microscopy in cases of tenofovir nephrotoxicity, that include mitochondrial depletion, and wide changes in mitochondria size and shape, with clumping, loss, and disorientation of cristae. In addition, mitochondrial injury may lead to apoptosis. Although tenofovir has not been studied, cidofovir is known to induce proximal tubular cell apoptosis by leading to caspase activation [11]. The mitochondrial pathway of apoptosis includes the release of mitochondrial proteins to the cytosol including cytochrome c (CytC), which is required for caspase 9 activation in the apoptosome Smac/Diablo, inhibitor of apoptosis proteins (IAPs), and apoptosis-inducing factor (AIF) that, among other actions, causes DNA injury. These are potential mediators of tenofovir-induced tubular cell injury that deserve further study. (c) Analytical and clinical consequences of tenofovir proximal tubular cell toxicity. Injured proximal tubular cells fail to perform their functions. These include reabsorbing low-molecular-weight proteins (such as vitamin D-binding protein (DBP) and *β*2-microglobulin) through the megalin-cubilin system (MCS), glucose through the sodium/glucose cotransporter 2 (SGLT2), aminoacids, phosphate and uric acid, secreting H+ and synthesizing calcitriol by the action of mitochondrial 1*α*-hydroxylase on 25(OH) vitamin D reabsorbed from the tubular lumenAs more immediate consequences we may observe a variable mixture of low-molecular weight proteinuria, glycosuria, aminoaciduria, hypophosphatemia, hypouricemia, renal tubular acidosis, and vitamin D insufficiency and even osteomalacia as a consequence both of insufficient calcitriol synthesis and urinary losses of 25(OH) vitamin D. Persistent tubular injury may promote tubular cell loss and eventual decreased glomerular filtration and renal failure.

Tenofovir is eliminated unchanged in the urine by a combination of glomerular filtration and proximal tubular secretion [8]. 20–30% of the drug is actively transported into renal proximal tubule cells by organic anion transporters (hOAT1, and to a lesser extent, OAT3) in the basolateral membrane [9, 10]. Subsequently the drug is secreted to the tubular lumen by the apical membrane transporters MRP-4 and MRP-2 (multidrug resistance proteins, encoded by ABCC4 and ABCC2 genes, resp.) [6] (Figure 2). A number of drugs interact with these transporters and may cause excessive entry or reduced outflow of the drug, favoring intracellular accumulation and increasing renal toxicity (Table 1).

Tenofovir has less adverse effects on blood lipids, fat accumulation, and mitochondrial toxicity than nucleoside phosphonate reverse transcriptase inhibitors [12]. Gastrointestinal symptoms are the most common side effects of tenofovir [12]. Kidney toxicity may lead to acute kidney injury (AKI), chronic kidney disease (CKD), and features of proximal tubular injury, including Fanconi syndrome, isolated hypophosphatemia, and decreased bone mineral density (Figure 2) [13–17].

## 2. Tenofovir Nephrotoxicity

Concerns regarding nephrotoxicity were initially raised by the structural similarity between tenofovir and the nephrotoxic acyclic nucleotide analogues adefovir and cidofovir. These two drugs cause a proximal tubulopathy, possibly in part due to decreasing mitochondrial DNA (mtDNA) replication through inhibition of mitochondrial DNA polymerase *γ* [3, 18]. However, only minimal mtDNA depletion was noted in renal proximal tubular cells cultured with tenofovir [19]. Furthermore, early randomized clinical trials and postmarketing data supported the renal safety of tenofovir in relatively healthy HIV+ individuals [7, 20]. Neither Fanconi syndrome nor drug discontinuation because of renal events was observed in early trials [12, 21]. However, case reports, observational studies, animal models, and even cell culture data support the notion that tenofovir is nephrotoxic for proximal tubular cells [22–27]. The mismatched results between clinical trials and case reports may be explained because clinical trials have strict inclusion and exclusion criteria. By contrast in routine clinical practice patients may have associated conditions, medications, or background that may predispose to tenofovir nephrotoxicity [22]. We now review the evidence for tenofovir nephrotoxicity and discuss potential molecular mechanisms and clinical approaches. 

## 3. Clinical Features of Tenofovir Nephrotoxicity

The main clinical presentations of tenofovir nephrotoxicity are (a) proximal tubular dysfunction with preserved renal function and (b) proximal tubular dysfunction associated with decreased renal function. Decreased renal function may be classified as AKI, CKD, or a glomerular filtration rate (GFR) that is decreased when compared with baseline values, albeit within normal limits. Currently available information suggests that all of them share a basic common pathogenesis and pathology, which will be discussed together.

Most reported cases of tenofovir-associated nephropathy identified a partial or complete Fanconi syndrome, associated or not with a reduction in GFR [20, 22, 32–35]. Fanconi syndrome is a generalized proximal tubulopathy. In its complete form it associates renal tubular acidosis, glycosuria with normoglycemia, aminoaciduria, hypophosphatemia, hypouricemia, and tubular proteinuria [6, 23] (Table 2). Tubular dysfunction may precede the decline of renal function. Tubular proteinuria implies the presence in urine of increased amounts of small-sized proteins that are freely filtered in the glomerulus but reabsorbed by proximal tubules. *β*2-microglobulinuria is prevalent among tenofovir-treated patients, even with normal GFR [36, 37]. Urinary *β*2-microglobulin is higher in patients with lower body weights, suggesting that it is indeed a consequence of tenofovir overdosing, and decreases upon tenofovir withdrawal [37]. Other manifestations of proximal tubulopathy in individual patients include osteomalacia and decreased bone mass due to phosphate wasting and/or calcitriol deficiency, since calcitriol is synthesized by mitochondria in proximal tubules [38–41]. Tenofovir causes bone toxicity in animal models, when given at 6–12 times higher dose than recommended for humans [42] but most studies comparing tenofovir with other antiretroviral regimes have not found significant differences in bone density between tenofovir-containing treatment and control subjects or the differences have been limited to certain bone sites [12, 43]. In this regard, it is conceivable that bone toxicity is secondary to moderate to severe proximal tubular dysfunction.

Tenofovir is associated with a small, but increased risk of AKI [22]. This is the most dramatic consequence of tenofovir nephrotoxicity. AKI may be observed even a few months after starting tenofovir in predisposed patients. Tenofovir-induced AKI is usually nonoliguric, but it may be oliguric, and may require dialysis [23, 44]. Evidence of proximal tubular dysfunction is usually present. After discontinuation of the drug, renal function usually recovers, at least partially. However, CKD requiring dialysis following AKI has been described in a patient treated with tenofovir and cidofovir [44]. 

The majority of studies did not find a significant higher risk of proteinuria, CKD, or end-stage renal disease (ESRD) requiring dialysis in HIV patients treated with tenofovir compared to those receiving other antiretroviral drugs [12, 43, 45–48]. This is somewhat expected since CKD is a severe, irreversible manifestation of kidney toxicity that may take many years to develop. CKD may be asymptomatic until GFR is <30 mL/min/1.73 m^2^. Thus, there is a real chance that nephrotoxicity might be overlooked, as serum creatinine may not raise above the upper limit of normal until GFR is <60 mL/min/1.73 m^2^. Patients should be trained to collect 24 h urine specimens for creatinine clearance calculation, since estimation of GFR based on serum creatinine by the Modification of Diet in Renal Disease (MDRD) or Cockroft-Gault formulae may underestimate the degree for renal dysfunction if muscle mass, as is frequently the case in HIV-infected individuals, is lower than their age and sex standards [49]. Some observational cohort studies describing low rates of renal dysfunction with tenofovir use were of short duration [11, 20, 22, 50]. A recent meta-analysis of 13 studies (>5767 patients) reported a significantly faster loss of kidney function (−5.4 mL/min) in patients receiving tenofovir compared with control subjects (mean difference between groups in GFR loss estimated by the Cockroft-Gault formula: 3.9 mL/min; 95% confidence interval (CI), 2.1–5.7 mL/min). However, a crucial piece of information, the time in which those 3.9 mL/min were lost, was missing [22]. A significantly smaller degree of renal function loss was reported in clinical trials than in observational studies (mean decrease in eGFR 1.5 versus 5.45 mL/min, resp.) [22]. Similar results, albeit nonsignificant due to the smaller number of studies, were observed when GFR was estimated by the MDRD formula [22]. In this regard, mean rates of eGFR loss as severe as −14.7 mL/min in less than 1 year (48 weeks) were reported in patients treated with tenofovir plus ritonavir-boosted protease inhibitor regimes and this was significantly greater than that in patients treated with tenofovir plus nonnucleoside reverse transcriptase inhibitor regimes or regimes without tenofovir (−4.5 mL/min) [8]. Declines in GFR averaging 7–10 mL/min/year have been reported in subjects treated with tenofovir [6]. This is not a modest rate of eGFR decline. In fact, this rate of decline is observed in diabetic or Fabry nephropathies and if maintained over time it will lead to ESRD in 10 years [51]. As a reminder the age-related estimated loss of GFR is −1 mL/min/year. Slower rates of decline in eGFR were observed in clinical trials of selected populations and unselected cohorts. However, some of the estimates reporting lower rates of GFR loss are not reliable due to a high rate of missing values during followup, which might have been biased by the loss of patients with nephrotoxicity [7]. For example, in a safety data analysis from France, Germany, and Italy values for serum creatinine were available at baseline for 2790 patients, but follow-up data were available only for 1704 patients: nearly 40% of patients lacked follow-up creatinine values [7].

Cardiovascular disease is now a leading cause of morbidity and mortality in HIV patients. This may be related to an increased prevalence of traditional cardiovascular risk factors as well as HIV-specific factors associated with antiretroviral therapy, chronic inflammation, and direct viral effects [52]. Tenofovir nephrotoxicity may impact cardiovascular risk by decreasing GFR and impairing the activation of vitamin D in proximal tubules. Both a decreased GFR and vitamin D deficiency are associated to increased cardiovascular risk [53, 54].

## 4. Incidence and Prevalence of Tenofovir Nephrotoxicity

Two studies have demonstrated tubular dysfunction with tenofovir in 17–22% of tenofovir-treated patients (versus 6 and 12% of HAART-treated or -naive HIV patients) [55, 56]. Some reports identified a trend toward higher incidence of hypophosphatemia in patients on tenofovir (incidence of serum phosphate <2 mg/dL: 16.7 per 100 person-years among tenofovir-treated patients versus 8.0 per 100 person-years in those without tenofovir; prevalence 9.8% among tenofovir-treated, 6.7% among nontenofovir, HAART-treated and 2.6% among treatment-naive, HIV-infected individuals) [55, 57] recognizing that hypophosphatemia is relatively common in HIV patients [58]. Glycosuria was found in 5 out of 7 nondiabetic patients biopsied for tenofovir nephrotoxicity with increased serum creatinine and residual diuresis [23]. This is a high percentage compared with the finding of non-diabetic glycosuria in 2% of tenofovir-treated patients with normal GFR [54], suggesting that dipstick glycosuria may be a cost-effective screening test for serious tenofovir-induced kidney injury.

The risk difference for AKI for tenofovir compared to control subjects was estimated to be 0.7% (95% CI 0.2–1.2%) in a recent metaanalysis [22]. 

A retrospective study of >1000 HIV-infected patients on tenofovir identified 1% whose sCr increased >120 *μ*mol/L [59]. A 4-year followup of 10,343 tenofovir-treated patients disclosed serious renal adverse events in 0.5% and an increase in serum creatinine ≥0.5 mg/dL in 2.2% of patients [7]. The SCOLTA observational study of 754 HIV-infected, tenofovir-treated patients found a 2.5% incidence of creatinine elevations over 1.5-fold the upper limit of normal in a mean followup of 19.5 months [60, 61]. The cut-off values used in these studies to define nephrotoxicity clearly underestimated this adverse effect. As a reminder, current AKIN criteria define AKI as an increment of serum creatinine of 0.3 mg/dL in 48 h [62]. Thus, an increase in serum creatinine but within normal limits (e.g., from 0.7 to 1.0 mg/dL) is already indicative of serious renal injury.

## 5. Pathogenesis

The proximal tubular cell is the main target of tenofovir toxicity due to its complement of cell membrane transporters that favor tenofovir accumulation. Current evidence suggests that mitochondria are the target organelles of tenofovir cytotoxicity. Proximal tubular cells account for most of the tubular transport of molecules, reabsorbing over 200 g ClNa, 1 kg glucose, and other molecules from the tubular lumen every day. Energy for this transport is provided by a high number of mitochondria. Proximal tubular cells are also rich in cell membrane transporters [63] (Figure 2). Proximal tubule mitochondria activate 25 dihydroxycholecalciferol by 1*α* hydroxylation, therefore yielding the active metabolite of vitamin D, calcitriol. Furthermore, they release the ammonia required by distal segments to secrete protons into the urine. Thus, mitochondrial injury will impair molecular transport, vitamin D activation, and urinary acidification (Figure 2). Mitochondria assembly needs the cooperation of both the nuclear and the mitochondrial genomes. Thus mitochondrial dysfunction may be the result of (a) mitochondrial genes that are defective or missing, (b) relevant nuclear genes that are defective or missing, or (c) normal assembled mitochondria that are damaged and lose function.

Tenofovir is an acyclic nucleotide phosphonate, like adefovir and cidofovir. Cidofovir is a recognized proximal tubular cell toxin, and the high incidence of toxicity prompted discontinuation of clinical trials for adefovir in the treatment of HIV infection [44, 64, 65]. Initial cell culture studies did not disclose significant tenofovir toxicity to human proximal renal tubules with the tests and culture conditions employed. Minimal mtDNA depletion and nonsignificant reductions in the mitochondrial protein cytochrome c oxidase were noted with tenofovir [3]. In this regard, tenofovir is considered a weaker inhibitor of mitochondrial DNA polymerase *γ* than adefovir and cidofovir [6]. However, more recent studies employing cell viability assays disclosed a 100-fold increase in cytotoxicity at 120 h in cells expressing OAT1 versus cells lacking the transporter [26] (Figure 2).

HIV transgenic mice exposed to tenofovir showed ultrastructural mitochondrial abnormalities and decreased proximal tubular mtDNA, but not optical microscopy changes [27]. No ultrastructural mitochondrial abnormalities were observed in control HIV transgenics or in tenofovir-treated wild-type (WT) mice. However, there were no differences in proximal tubule mtDNA between HIV transgenics and WT, either in the presence or absence of tenofovir. This observation does not support the hypothesis that mtDNA depletion, a complication of HIV infection, might prime for more severe mitochondrial dysfunction if exposed to tenofovir [66]. 

Rather, additional factors related to HIV infection might be relevant. In the absence of HIV, rats exposed to tenofovir developed proximal tubular dilatation, ultrastructural mitochondrial abnormalities, depleted mtDNA, depressed respiratory chain enzyme expression [24], and specific downregulation of proximal tubular sodium-phosphorus cotransporter, sodium/hydrogen exchanger 3, and aquaporin 2 [25]. Morphological evidence of mitochondrial toxicity was also found in human biopsies of tenofovir nephrotoxicity [23]. Tenofovir is an adenosine analogue, like didanosine. In contrast to didanosine, which induced significant hepatic mtDNA depletion in rats and mice, tenofovir had no liver effects, confirming the importance of specific proximal tubular transporters in increasing the intracellular tenofovir concentrations to toxic levels in proximal tubules [24, 27]. 

The extent to which mitochondrial damage is responsible for proximal tubular cell loss and the mechanisms of such an effect remain unclear. Inhibition of mtDNA polymerase *γ* encoded by *POLG* has been proposed to have a central role in tenofovir-related mitochondrial toxicity [67]. Inherited *POLG* abnormalities lead to decreased mtDNA content and accumulation of mtDNA defects [68]. Depletion of mtDNA may lead to fatty acid and dicarboxylic acid accumulation, lacticacidosis and ROS damage, and both resistance and sensitivity to apoptosis [69–73]. However, in nucleoside reverse transcriptase inhibitor-induced lipodystrophy, mtDNA-dependent mitochondrial functions are preserved despite severe mtDNA depletion and the presence of apoptosis did not correlate with mtDNA content [71].

Damage to mtDNA has been well studied in adefovir nephrotoxicity [64]. Renal biopsies from patients with adefovir-induced AKI revealed proximal tubule necrosis containing dysmorphic and enlarged mitochondria, deficiency of mtDNA encoded enzymes, and a 30–60% reduction in mtDNA in injured tubules. However, the in vitro toxicity index based on the relative rates of mtDNA and nuclear DNA replication underpredicts the toxicity of some drugs, suggesting that factors other than inhibition of DNA polymerase *γ* be responsible for nephrotoxicity [74]. Kidney mtDNA depletion was associated with HIV infection and concurrent tenofovir/didanosine therapy but not to tenofovir use alone, while kidney ultrastructural mitochondrial abnormalities were seen with tenofovir use [75]. 

Interestingly, mitochondria are key organelles in apoptotic cell death [76]. In this regard, cidofovir induced OAT1-dependent, probenecid-sensitive, and caspase-dependent apoptosis specifically in proximal tubules [44]. Whether tenofovir activates similar pathways should be studied (Figure 2(b)).

## 6. Pathology

There are several reports on the underlying pathology of human tenofovir nephrotoxicity [23, 75, 77]. Detailed renal pathology of cases biopsied for AKI or for proteinuria and mild renal dysfunction were recently provided [23]. The major renal biopsy finding was proximal tubular injury, ranging from diffuse and severe to mild and localized. This was associated with varying degrees of chronic tubulointerstitial scarring (i.e., tubular atrophy and interstitial fibrosis) [23]. Either findings of acute tubular injury or findings of chronic injury may predominate, leading to a diagnosis of acute or chronic tubulointerstitial nephropathy, respectively. By light microscopic proximal tubular changes resembled toxic acute tubular necrosis and included luminal ectasia, irregular luminal contours, prominent nucleoli, and loss of brush border. A distinctive finding was prominent eosinophilic intracytoplasmic inclusions representing giant mitochondria, as confirmed by ultrastructural studies [23]. In some proximal tubular cells, the number of mitochondria was markedly reduced, consistent with mitochondrial depletion. Mitochondria varied widely in size and shape. Many enlarged mitochondria displayed prominent clumping, loss, and disorientation of cristae. Kidney mtDNA depletion was associated with HIV infection and concurrent tenofovir/didanosine therapy but not tenofovir use alone, while kidney ultrastructural mitochondrial abnormalities were seen with tenofovir use [74]. 

In tenofovir-treated mice no disruption of glomeruli or proximal tubules was observed by light microscopy. Ultrastructurally in proximal tubules from tenofovir-treated HIV transgenic mice but not from wild-type mice included there was an increased number and irregular shape of mitochondria with sparse fragmented cristae [27].

## 7. Prediction, Prevention, and Treatment of Nephrotoxicity

The overall safety profile of tenofovir is quite positive. Thus, a prediction of who is at risk of nephrotoxicity is required to adequately manage those patients. Multivariate analysis of postmarketing clinical data showed that advanced age, low body weight, higher serum creatinine levels before starting tenofovir treatment, comorbidities (diabetes, hypertension, HCV coinfection) concomitant nephrotoxic medications, advanced HIV infection (low CD4 counts, AIDS), and, in some studies, male sex were risk factors for tenofovir-induced GFR reduction [7, 8, 61] (Table 3).

The odds of developing significant renal function reduction were 3.7 times higher for patients receiving tenofovir plus ritonavir-boosted protease inhibitor regimes than for those receiving tenofovir plus nonnucleoside reverse transcriptase inhibitor-based therapy, even adjusting for HIV load [8]. Underlying renal disease with low GFR enhances the risk for tenofovir toxicity by decreasing tenofovir renal clearance and increasing the amount of tenofovir in the circulation and proximal tubular cells [78]. Dose reduction is indicated if GFR is low, but this may be difficult to implement when a single pill contains several antiretrovirals. Certain ABCC2 gene (encoding the outward tenofovir transporter MRP2) polymorphisms were associated with tenofovir-induced renal dysfunction [56].

Recent guidelines from the HIV Medicine Association of the IDSA recommend at least biannual monitoring of renal function, serum phosphorus, proteinuria, and glycosuria in HIV patients receiving tenofovir with GFR <90 mL/min/ 1.73 m^2^, other comorbid diseases or cotreated with protease inhibitors, due to the potential risk of nephrotoxicity [22, 28, 79, 80]. Mild cases of tenofovir-associated nephropathy may be detected testing urine for features of proximal tubule injury and by measuring bone density [22]. Urinary features of proximal tubular dysfunction include glycosuria, the presence of increased amounts of low-molecular-weight proteins such as *β*2-microglobulin or light chains, aminoaciduria, and inappropriate amounts of uric acid or phosphorus, coupled with a reduced phosphate reabsorption rate. In addition serum uric acid and phosphate are low and serum bicarbonate may decrease (Table 2).

The most effective treatment of tenofovir nephrotoxicity is stopping tenofovir. Features of nephrotoxicity frequently improve following discontinuation of the drug. In a followup of 20 ± 26 months after discontinuation of tenofovir following AKI, roughly 50% of patients completely recovered renal function to baseline levels, including a patient who had required dialysis for 4 months [23]. Other patients had partial recovery of renal function, from a mean peak sCr 5.6 ± 3.8 to sCr 1.5 ± 0.3 mg/dL. Renal function and features of proximal tubular dysfunction improve over weeks to months [13, 81] (Table 2). Early detection of nephrotoxicity and tenofovir withdrawal are key to avoid irreversible tubulointerstitial damage.

## 8. Future Developments: towards Nephroprotection

Theoretically nephroprotection could be achieved by preventing tenofovir entry into proximal tubular cells, facilitating its exit or administering drugs that protect tubular cells from injury. Probenecid, an inhibitor of hOAT1, is used to prevent cidofovir nephrotoxicity and may also protect from tenofovir [29, 30, 66]. However, 56% of patients had side effects ascribed to probenecid when used to prevent the proximal tubular toxicity of cidofovir, which were dose limiting in 7% [31]. Rosiglitazone, a peroxisome proliferator-activated receptor-gamma agonist that induces the expression of many proximal tubular cell transporters, protected rats from tenofovir-induced renal failure and proximal tubular dysfunction [25]. However, concerns over the cardiovascular safety of rosiglitazone have led to its withdrawal from European markets [82]. Clearly more research is needed on nephroprotective strategies using drugs.

Another approach is to modify the tenofovir molecule to decrease proximal tubular uptake. Low oral bioavailability, renal toxicity, and poor cell penetration are limitations of acyclic nucleotide phosphonates. These undesirable features can be eliminated by esterifying the compounds with an alkoxyalkyl group, in effect disguising them as lysophospholipids [2]. Among other advantages, in this modified form, drugs are not recognized by the transport mechanisms that cause their accumulation in renal proximal tubular cells. As a consequence, they lack nephrotoxicity in rats. A member of this class of molecules, hexadeciloxypropyl-tenofovir (CMX157), is now in clinical development [83]. In addition, novel ribose-modified NtRTI are currently being evaluated in the clinic. These molecules are less efficiently transported into and less cytotoxic to proximal tubular cells than acyclic nucleotides [26]. These include GS-9148 and its oral prodrug GS-9131 [26]. Hopefully the clinical development of any of these strategies will result in the availability of less cytotoxic albeit effective NtRTI drugs.

## 9. Summary

Tenofovir nephrotoxicity is characterized by proximal tubular cell injury. This may result in partial or complete Fanconi syndrome, AKI or CKD. Drug withdrawal is the main therapeutic option. This usually results in improvement of clinical manifestations of kidney injury, which may be only partial. Thus, prevention of nephrotoxicity by careful monitoring of high-risk populations is paramount. Proximal tubular cells are particularly sensitive to the toxic effects of tenofovir due to their unique set of cell membrane transporters that favor entry of the drug. In this regard, the design of novel, less cytotoxic drugs is centered on chemical modifications that limit entry into proximal cells. Mitochondria are targets of tenofovir cytotoxicity. However the precise molecular mechanisms of injury are unclear.

## Figures and Tables

**Table 1:** Drugs interfering with proximal tubular tenofovir transporters.

Transporter	Drug interaction	Effect
hOAT1	Probenecid inhibits hOAT1 NSAIDs inhibit hOAT1 Acyclovir DDI competes with tenofovir	Probenecid decreases the incidence of renal toxicity by cidofovir, might for tenofovir [28] Acyclovir increases serum concentrations of tenofovir Tenofovir increases DDI levels [29]

MRP-4	Inhibition of MRP-4: probenecid dipyridamole NSAIDs [30]	Acyclovir increase serum concentrations of tenofovir NSAIDs associated with tenofovir nephrotoxicity [30, 31]
	Cidofovir, acyclovir, valaciclovir, ganciclovir, and valganciclovir	

MRP-2	Ritonavir is transported by MRP-2	Ritonavir increases tenofovir concentration and has been associated with tenofovir nephrotoxicity

**Table 2:** Biochemical features and time course of a case of tenofovir-associated Fanconi syndrome.

	Before tenofovir	After 4 years of tenofovir	One month after tenofovir	Five months after Tenofovir
Serum				
Creatinine (mg/dL)	0.96	1.5	1.3	0.9
Glucose (mg/dL)	97	88	94	79
Uric acid (mg/dL)	8.4	1.2	1.8	3.4
Potassium (mmol/L	4.2	4.5	4.1	No data
Calcium (mg/dL)	9.1	8.5	9.2	9.2
Phosphorus (mg/dL)	3.8	2	3.2	No data
Total proteins (g/dL)	No data	7.8	8.5	7.9
PTH (pg/mL)	No data	128	55	27
Alkaline phosphatase (UI/L)*	No data	271	242	154
Urine and calculated parameters				
Glycosuria (mg/dL)	Negative	1000	No data	Negative
Proteinuria (mg/dL)	Negative	50	53	Negative
Tubular reabsorption of phosphate (%)	No data	34	59	
Creatinine clearance (mL/min)	86	26	40	No data
Albuminuria/creatinine (mg/g)	10	937	303	No data
Light chains *κ*/*λ* (mg/dL)	No data	4.2/4.1	2.5/1.7	0.6/<0.4
ß-2 microglobulin (ug/24 h)	No data	4	4	No data

GGT<10 UI/L at all time points.

**Table 3:** Predictors of significant renal function decline.

Preexisting renal impairment	
Older age	
Advanced HIV disease	
Vasculometabolic disease	
Concomitant use of nephrotoxic drugs or protease inhibitors	
Low body weight	
ABCC2 gene (encoding the outward tenofovir transporter MRP-2) polymorphisms	
